# The vascular-immune-neural network: a new pathophysiological paradigm and the dawn of cytoprotection in stroke

**DOI:** 10.1016/j.ebiom.2025.105843

**Published:** 2025-07-05

**Authors:** Yiliang Fang, Zhiyi Zhu, John H. Zhang, Fu-Dong Shi, Qingwu Yang

**Affiliations:** a Department of Neurology, Second Affiliated Hospital of Army Medical University (Xinqiao Hospital), No. 183 Xinqiao Main St, Shapingba District, Chongqing 400037, China; b Chongqing Institute for Brain and Intelligence, Guangyang Bay Laboratory, Chongqing 400064, China; c Department of Neurosurgery, Loma Linda University, Loma Linda, CA 92350, USA; d Department of Neurology, China National Clinical Research Center for Neurological Diseases, Beijing Tiantan Hospital, Capital Medical University, Beijing 100070, China; e Department of Neurology, Tianjin Neurological Institute, Tianjin Medical University General Hospital, Tianjin 300052, China

**Keywords:** Acute ischaemic stroke (AIS), Vascular-immune-neural network (VIN), Immune response, Cytoprotective therapy

## Abstract

At present, no drugs related to brain protection have received strong recommendations from the guidelines in the treatment of stroke. Given the increasing understanding of the interactions between the immune system and the central nervous system and the promising efficacy of our latest immune therapy, it is necessary to reconsider the critical role of immunity in the pathophysiology of stroke. In this review, we propose a new pathophysiological paradigm, namely, the vascular-immune-neural network (VIN), emphasizing the dynamic and continuous interactions between the immune system and the vascular neural network. We update current pathways of brain–immune communication during AIS and dissect the panorama of the immune response to stroke, both in the global brain and the whole body, highlighting the importance and features of the VIN paradigm in stroke. Additionally, we summarize recent advances in immune-related therapies to shed light on the use of cytoprotective treatments throughout the stroke process.

## Introduction

Stroke is the leading cause of disability and the second leading cause of death worldwide.^1^ Acute ischaemic stroke (AIS), which is caused primarily by an interruption in cerebral blood flow, leads to the rapid death of neurons due to the extreme intolerance of these cells to oxygen and glucose deprivation. Despite the demonstrated efficacy of numerous drug candidates in preclinical trials, the overwhelming majority of these drugs ultimately fail in subsequent steps of clinical development.^2^ This translational gap can be attributed to multifaceted issues, including the low quality of preclinical studies, assessment bias of exploratory research, delayed initiation of treatment, insufficient integration with reperfusion therapies,^3^^,^^4^ and incomplete elucidation of the pathophysiology of stroke.

The proposal of the neurovascular unit (NVU) concept by a National Institutes of Health workshop in 2001 was pivotal for understanding the neurovascular interactions that regulate cerebral blood flow, the pathophysiological process of stroke, and the interpretation of blood oxygen level-dependent magnetic resonance imaging (MRI) signals.^5^ This concept is a useful framework for investigating how the structural and functional interplay between brain cells and the microvasculature coordinates responses to injury.^5^ Evolutionarily, an advanced stroke pathophysiology paradigm has been proposed; this paradigm, namely, the vascular neural network (VNE), includes cerebral vessels both upstream and downstream of the NVU that control perfusion and reperfusion.^6^ The VNE emphasizes the critical roles of vascular smooth muscle, endothelial cells and perivascular innervation in stroke prevention and reperfusion. Unfortunately, both the NVU and VNE overlook the critical role of immune cells in the pathophysiology of stroke, possibly because the brain considered to be in a state of “immune privilege” under physiological conditions.

Circulating immune cells rapidly respond to tissue injury, including stroke. Consequently, the activation of the immune response occurs almost simultaneously with the occlusion of cerebral vessels, followed by interaction with vascular endothelial cells; these phenomena enable immune cells to extravasate across the blood‒brain barrier (BBB) to reach the brain parenchyma. Then, egressed immune cells remain in the global brain for a long period of time and ultimately influence many other organs.^7^^,^^8^ In recent years, the critical roles of inflammation in stroke—ranging from increasing risk of stroke^9^ to contributing to acute inflammatory injury and chronic repair^10^—have been increasingly recognized. Additionally, immunomodulation has emerged as a highly promising approach for treating stroke.^11^, ^12^, ^13^, ^14^ In this review, we propose a new pathophysiological paradigm of stroke—the vascular immune neural network (VIN)—to highlight the critical importance of interventions that target the immune response in stroke treatment.

## Pathways of NVU cell–immune cell communication

**Fig. 1:**
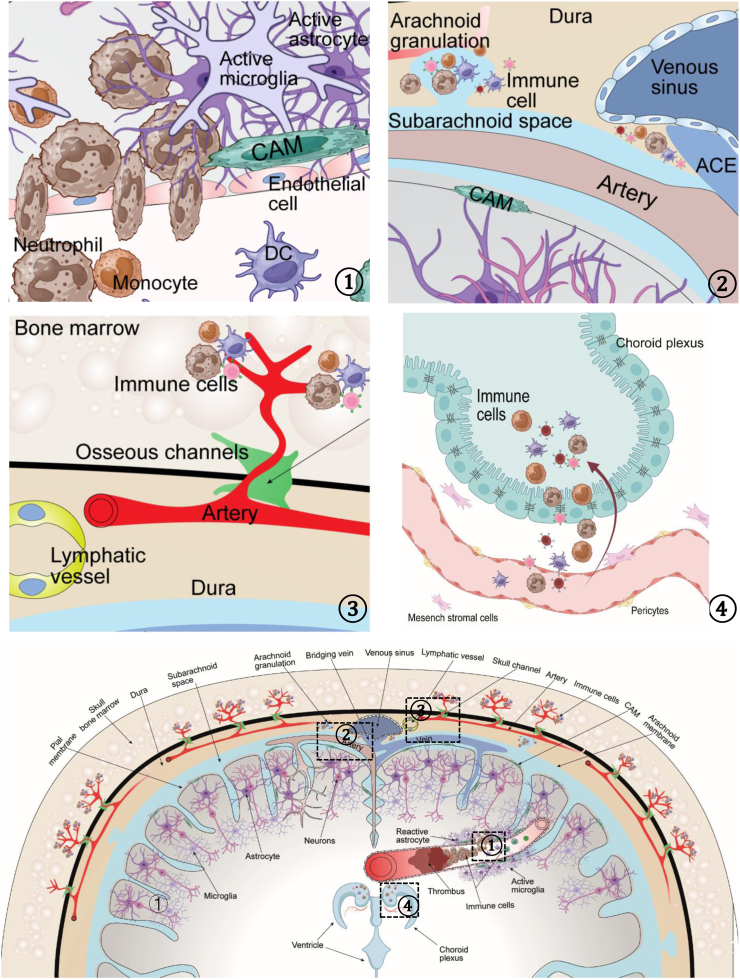
Pathways of NVU cell–immune cell communication. Four distinct neuroimmune trafficking pathways (top, detailed pathways; bottom, overview). Paracellular and transcellular endothelial transmigration ①: Immune cells cross the blood‒brain barrier (BBB) via either tight junction (TJ) disruption (paracellular route) or vesicular transport through endothelial cells (transcellular route). Immune cells traverse the arachnoid granulations or arachnoid cuff exit (ACE) points to enter the subarachnoid cerebrospinal fluid (CSF) compartment. ②; Intradural lymphatic vessels and the osseous channel pathway provide conduits for immune cell trafficking into meningeal spaces ③; immune cells extravasate across the choroid plexus epithelium into the ventricular CSF ④.

Since the precise interactions between the brain vasculature and the central nervous system are described in the NVU^15^ and VNE,^6^ this review focuses on the interactions between immune cells and the central vascular system or the nervous system during stroke (Fig. 1).

The immune system, which is somewhat comparable to the vascular and nervous systems, is a pivotal component of the brain. Brain-resident immune cells, such as microglia and CNS-associated macrophages (CAMs), maintain direct connections with neurons or cerebrovascular cells. Microglia not only detect damage and phagocytose debris during CNS development and disease but also secrete proinflammatory cytokines into the cerebrovascular lumen, contributing to peripheral immune cell infiltration.^16^^,^^17^ Microglia can also remodel neuronal circuits and perform neuroprotective functions.^16^^,^^18^ Single-cell data have shown that after stroke, activated microglia exert protective effects through phagocytosis and promote neuronal regeneration while exacerbating brain damage by releasing inflammatory factors.^18^^,^^19^ CAMs are important checkpoints at CNS gateways, and they regulate the drainage of CNS antigens and facilitate immune alarm exchange with the CNS.^17^ CAM numbers also increase within the meninges and perivascular spaces of the ischaemic hemisphere during the acute phase of stroke and progressively accumulate in the meninges proximal to the ischaemic lesion in the chronic phase after stroke.^20^ However, the specific roles of brain-resident immune cells during stroke require further investigation.

Peripheral immune cells that enter the CNS must pass through the BBB, which is a physical and functional barrier that maintains CNS “immune privilege” by strictly controlling the entry and exit of blood cells^21^ During healthy immune surveillance, immune cells need to participate in a series of interactions with cerebrovascular endothelial cells to extravasate through tight junctions (TJs) into the brain parenchyma.^21^ Considering that complex and continuous TJs make rapid diapedesis difficult, immune cells can rapidly traverse the inflamed BBB via transcellular pathway under conditions of insult, such as stroke.^21^ This mechanism enables immune cells to enter the brain parenchyma through pores that form on the luminal side of the cerebrovascular endothelium without disrupting the morphological integrity of TJs. Immune cells can also extravasate into cerebral spinal fluid in the choroid plexus by crossing fenestrated endothelium cells or in the subarachnoid space by crossing the blood‒meningeal barrier. ischaemic stroke disrupts the integrity of the blood–CSF barrier, creating a key route for T cell invasion.^22^

Furthermore, in addition to the pathway that depends on the vascular network, peripheral immune cells can access the CNS through alternative routes. Recently, the discovery of intradural lymphatic vessels^23^ and osseous channels (so-called ‘skull channels’)^24^ revealed new pathways through which immune cells access the meninges. In humans, two channels allow immune cells to travel from the dura mater to the subarachnoid CSF^25^; that is, immune cells can cross the arachnoid granulation or the newly defined arachnoid cuff exit (ACE) points, which are structures within discontinuities in bridging veins where immune cells can cross the arachnoid barrier.^25^ In mice, only the ACE pathway is present.^25^ In murine stroke models, brain-infiltrating CD14^+^ neutrophil subpopulations are more likely to come from the bone marrow of the skull through skull channels than from the distant tibia.^24^ However, whether the permeability of ACE points changes during stroke to facilitate immune cell migration requires further investigation. Moreover, the mechanisms that drive immune cell migration through skull channels, as well as the specific functions and underlying processes, remain unclear.

In conclusion, peripheral immune cells can infiltrate the cerebral parenchyma through multiple pathways during different phases of stroke, thereby exerting significant effects. During the acute phase, a large number of immune cells enter the brain; myeloid cells infiltrate the infarct core region, whereas natural killer (NK) cells are primarily distributed in the peri-infarct area.^26^ The innate immune response gradually decreases, whereas the adaptive immune response becomes activated during the subacute phase.^8^ Peripheral lymphocytes undergo activation and initiate infiltration into the infarct core. In contrast to innate immune cells, lymphocytes persist during the chronic phase; rather than receding, they continue to spread in the brain parenchyma and eventually fill the entire brain several years later.^7^

## The panorama of the immune response in stroke

The immune response functions throughout the whole body and across the entire course of stroke, although the type and intensity of this response vary significantly across various stages and locations.

## Acute phase (0–24 h): initiation of the immune response

### Hypoxic phase (0–4.5 h): vascular–immune interactions sound the horn of immune response

Hypoxia is one of the most important hallmarks of stroke, as it not only damages neurons in the brain but also increases BBB permeability and reprograms the immune response. Hypoxia has been demonstrated to disrupt the BBB by inducing the redistribution of membranous claudin-5 into the cytosol^27^ or by causing pericytes to detach from vessel walls to loosen the intracellular junctions between endothelial cells.^28^ In response to hypoxic insult, neutrophils are rapidly activated to release proinflammatory factors such as TNFα, form neutrophil extracellular traps, and increase the expression of CD11b/CD18, which bind to β2 integrins on endothelial cells.^29^^,^^30^ The neutrophils and monocytes that accumulate in the early stages of stroke can rapidly deplete oxygen in the microenvironment, further exacerbating hypoxia in the infarct core area.^30^ Similarly, in vitro data have shown that hypoxia alone is sufficient to recruit human monocytes to the hypoxic core region^31^ and promote macrophage polarization toward the M1 phenotype.^30^ Resident microglia are also quickly activated by hypoxia, and these cells become hypertrophic before neuronal cell death occurs to produce proinflammatory cytokines and chemokines.^32^

### Injury phase (4.5–24 h): immune cells are recruited to induce true inflammatory injury in neurons

Under conditions of prolonged glucose and oxygen deprivation, neurons die first. Gasdermin E, which is a pyroptotic executor, is widely expressed in both mouse and human neurons.^33^ Consequently, vascular occlusion often results in neuronal necrosis or pyroptosis,^33^ which cause the release of a large numbers of DAMPs, such as high mobility group protein B1,^34^ and large quantities of neuronal antigens, myelin antigens, and nuclear-, glial-, and endothelial-derived peptides.^35^ Both brain-derived DAMPs and antigens disseminate systemically via the bloodstream or lymphatic system at very early time points (within 3 h), and their circulating concentrations are positively correlated with the severity of cerebral damage.^36^^,^^37^

DAMPs activate brain-resident and peripherally derived innate immune cells, such as NK cells, and recruit these cells to migrate into the infarct area along a concentration gradient. Peripherally, immune cells are recruited from both peripheral immune organs and central bone marrow reservoirs,^38^ demonstrating that infiltrating immune cells migrate from two origins. For example, the rapid upregulation of CXCL1, which is a ligand of CXCR2, and granulocyte-colony stimulating factor (G-CSF) in the bloodstream after cerebral ischaemia induce the early (within 4 h) release of CXCR2-positive granulocytes from the bone marrow.^39^ These DAMPs further exacerbate sterile inflammation via pattern recognition receptors (PRRs), such as Toll-like receptor (TLR) 2/4, NOD-like receptor protein 3 (NLRP3), and cyclic GMP-AMP synthase-stimulator of interferon genes (cGAS-STING), and trigger a cytokine storm in the core infarct area, thereby initiating true inflammatory injury.

Although inflammatory effects are two-sided, once a cytokine storm occurs, it becomes highly destructive and potentially fatal. This inflammatory “fire” exacerbates BBB damage and reduces the penumbra.^10^ Therefore, timely and appropriate control of cytokine storms in the brain after stroke may be significant for alleviating the pathological development of stroke.

## Subacute phase (1 day–7 days): the vascular neural network spreads the immune response of the brain to the whole body

Interestingly, innate immune cells mediate proinflammatory “fires” in the brain, whereas adaptive immune cells exhibit a “ice” state of immunodepression in the periphery.^8^ Although the inhibition of the peripheral immune response is believed to prevent excessive inflammatory damage to the brain, the detailed mechanisms are still unclear, particularly the mechanisms underlying the selective suppression of adaptive immune cells.

Lymphopenia is a well-established contributor to immunodepression after stroke.^40^ Mice with middle cerebral artery occlusion (MCAO) exhibit marked reductions in circulating lymphocyte counts accompanied by splenic and thymic atrophy^40^; this phenomenon has been corroborated in AIS patients.^26^ Stroke also induces the contraction of NK cell numbers in the periphery and the suppression of NK cell responses in the brain.^26^ The shift in T helper (T_H_) cell functional polarization in peripheral lymphoid organs further exacerbates stroke-induced immunodepression. Poststroke Treg expansion occurs not only in the brain^41^ but also in peripheral lymphoid tissues, the circulation, and the BM.^19^^,^^42^ Concurrently, stroke induces Th cell cytokine dysregulation, which is characterized by suppressed interferon-γ (IFN-γ) production and elevated interleukin-4 (IL-4) production by CD4^+^ T cells.^40^

Mechanistically, proinflammatory factors that are released due to ischaemic brain damage not only activate the hypothalamic‒pituitary‒adrenal (HPA) axis but also increase sympathetic nerve system (SNS) activity, which results in brain-mediated immunodepression in the periphery.^26^^,^^40^ Released catecholamines can not only promote Treg cell proliferation and immunosuppressive capacity via β2-adrenergic receptor-dependent activation of protein kinase A (PKA) signalling^43^ but also induce lymphocyte and NK cell apoptosis to cause lymphodepletion. However, clinical data have shown that the use of β2-adrenoreceptor blockers does not affect the severity or outcome of ischaemic stroke, but the effects on the frequency of pneumonia remain controversial.^44^ Paradoxically, in spite of the lymphodepletion that occurs in peripheral lymphoid organs, stroke triggers the accumulation of total T cells, regulatory T cells (Tregs), and activated B cells in the bone marrow (BM).^39^^,^^42^ This indicates that, in addition to apoptosis, the atrophy of peripheral immune organs and the thymus might also be caused by the redistribution of immune cells into the BM. However, this hypothesis requires further empirical validation. Therefore, the exact mechanism by which stroke induces immunodepression in the periphery is still unclear. Moreover, although the release of large amounts of autoantigens is detected after stroke, the effects of these autoantigens on immune responses have rarely been studied.

## Chronic phase (longer than 14 days): lasting impact of the immune response on the whole body

The immune system plays a critical role during the chronic recovery phase of stroke because of its memory properties and the delayed initiation of the adaptive immune response. Therefore, elucidating the function and mechanisms of the immune response in the chronic phase may provide many promising therapeutic targets for neuronal protection against stroke.

**Fig. 2:**
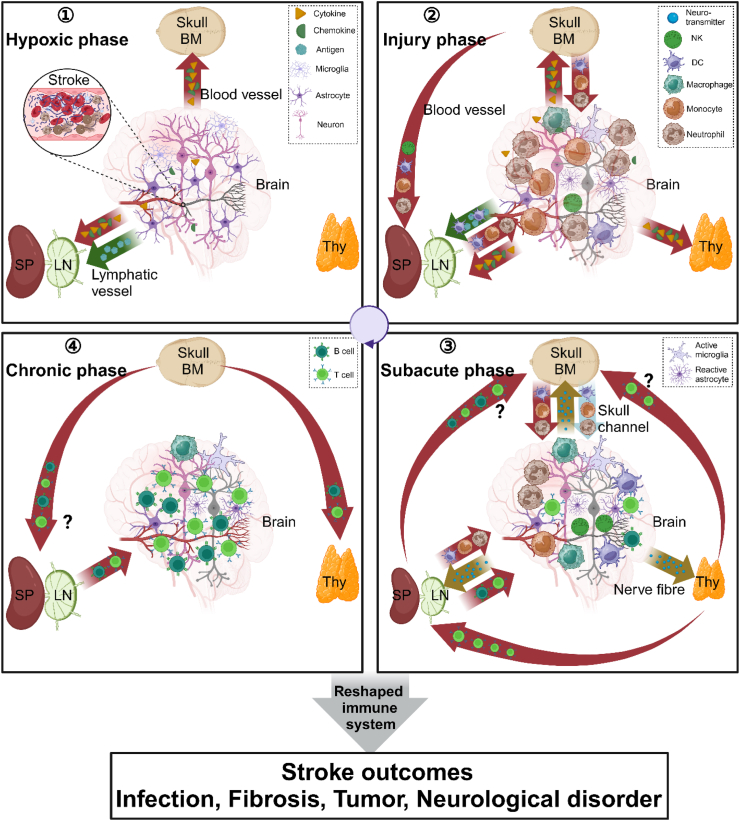
The panorama of the immune response in stroke. Only selected elements are shown. Upon the occlusion of cerebral vessels, pyroptotic neurons release antigens and damage-associated molecular patterns (DAMPs) while activated immune cells secrete proinflammatory cytokines and chemokines into systemic circulation ①. This chemotactic signalling regulates the recruitment of peripheral and bone marrow-derived innate immune cells into the brain parenchyma ②. Concurrently, the bone marrow initiates a compensatory redistribution of myeloid precursors to replenish peripheral lymphoid organs ②. As neuronal apoptosis progresses and neuroinflammation intensifies, central neuroimmune pathways become activated (such as the release of neurotransmitters), driving splenic and thymic atrophy through lymphocyte apoptosis or redistribution to the BM ③. In the chronic phase of stroke, lymphocytes continuously infiltrate into the CNS parenchyma ④. Simultaneously, bone marrow-infiltrated T cells recirculate to repopulate peripheral lymphoid compartments ④. Figure were prepared on biorender.com.

After the chronic phase begins, peripheral immunosuppression and inflammatory responses in the brain are relieved. The atrophic thymus and spleen as well as the degree of lymphocytopenia return to normal within approximately two weeks.^26^ However, there is still a lack of research on the mechanism underlying such rapid recovery, and it is not clear whether immune cells that enter the bone marrow reenter the periphery during the chronic phase. Although the inflammatory storm in the brain subsides, the numbers of adaptive immune cells, especially Treg cells and T helper 1 (T_H_1) cells, continue to increase and persist in the brains of stroke patients for several months^41^ or even decades (Fig. 2). Substantial evidence suggests that both Foxp3^+^ regulatory T (Treg) cells and effector CD4^+^ T cells can perform neuroprotective functions.^41^^,^^45^ Treg cells in the brain can suppress neurotoxic astrogliosis by producing amphiregulin, which is a low-affinity ligand of epidermal growth factor receptor, to enhance neurological recovery.^41^ However, naturally occurring Treg cells can also suppress the myelin-specific T cell-mediated protective immune response, potentially impeding the repair of myelinated axons.^45^^,^^46^ Additionally, certain self-reactive CD4^+^ T cells clones that exhibit T_H_1-like phenotype can promote recovery after CNS injury, partly by modulating myeloid cells through IFN-γ.^45^ In contrast, the persistent infiltration of CD8^+^ T cells^47^ and B cells^48^ is generally considered deleterious. Nonetheless, specific subtypes, such as regulatory B cells,^49^^,^^50^ macrophage-like B cells^51^ and regulatory-like CD8^+^ T cells,^52^ have been demonstrated to exert beneficial effects in mouse models of stroke. Therefore, the influence of adaptive immune cells with long-term memory properties is highly subset-specific and time-dependent.

After stroke, the resulting insults reshape blood vessels and the immune system, which, in turn, impacts other organs throughout the body. Both Liesz et al. and Liu et al. revealed that brain ischaemia increased the levels of the circulating alarmin HMGB1 protein and the Notch1 ligand DLL1,^53^ leading to persistent activation of vascular endothelial cells across the body for up to four weeks poststroke. This process exacerbates atheroprogression in systemic vessels. Chronic poststroke effects on systemic immunity also increase nonspecific responsiveness via epigenetic, transcriptional, and metabolic programs; this phenomenon is called “trained immunity”.^54^ The release of IL-1β after stroke induces persistent proinflammatory changes in monocytes/macrophages, causing multiorgan damage, most notably myocardial fibrosis and dysfunction, in both mice with MCAO and stroke patients.^54^ The release of CNS-derived antigens can trigger the activation of antigen-specific T and B lymphocytes. In addition to exacerbating stroke outcomes through neuroinflammatory cascades, these cells also promote aberrant autoantibody production, thereby contributing to the onset or progression of systemic autoimmune disorders.^55^ Therefore, the chronic effects of stroke are far-reaching and long-lasting, contributing to the development of other conditions, such as cancer^56^ and autoimmune diseases.^57^

## The vascular immune neural network (VIN)

Due to the concept of CNS “immune privilege”, the critical role of immune cells during the brain injury phase has long been overlooked by researchers. However, in-depth studies of the fine structure of the brain have revealed the presence of not only lymphatic vessels in the CNS but also immune pathways that connect the bone marrow of the skull directly to the dura mater.^24^^,^^25^ Nevertheless, the importance of immune cells has not been recognized in previous pathophysiological theories of stroke.^5^^,^^6^ As mentioned earlier, with the discovery of multiple channels for neuroimmune interactions, the extensive infiltration of immune cells after vascular infarction and the pivotal role of immune cells in the entire pathological process of stroke, we propose the VIN as a new framework to update the NVU and VNE paradigms.

The VIN paradigm highlights the continuous and dynamic involvement of the immune system in stroke pathology. From the acute onset to the recovery phase of stroke, the immune system plays pivotal roles in conjunction with the VNE. Even prior to stroke onset, various factors can modulate stroke outcomes by influencing immune responses. Hypertension, an independent and well-established risk factor for stroke, exacerbates stroke risk and worsens prognosis by promoting immune cell infiltration into the brain, partly through upregulation of intercellular adhesion molecule-1 (ICAM-1).^9^ Similarly, diabetes contributes to a pro-inflammatory state, which may aggravate acute stroke outcomes.^58^ Moreover, immune mechanisms underpin complex bidirectional interactions between the brain and heart. In particular, inflammation is a critical mediator in both the pathogenesis of cardioembolic stroke and the development of stroke-induced cardiac dysfunction.^59^

**Fig. 3:**
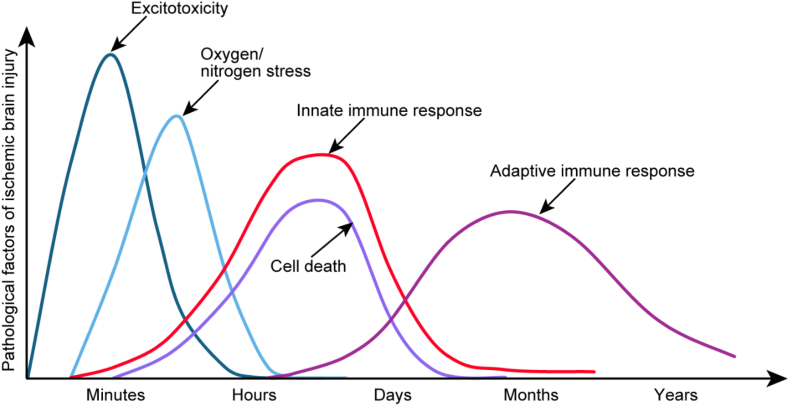
Pathological factors associated with ischaemic brain injury at different time points after stroke. Upon cerebral vessel occlusion, excitotoxicity and oxidative/nitrosative stress rapidly escalate, peaking and subsequently diminishing within a short period. Concurrently, the innate immune response is activated and intensifies, correlating with an increase in neuronal death. As the innate immune response wanes, the adaptive immune response persists, often enduring for months to years.

Blood vessels not only transport oxygen and nutrients to the brain but also transport immune cells to the brain. Vascular infarction is accompanied by hypoxia and the initiation of an immune response; therefore, we believe that immune intervention after stroke should be done as early as possible, before vascular therapy, in an ambulance or even at home. As neuronal death increases, innate immune cells continue to infiltrate and become activated, and these processes peak over several days. After the resolution of the innate immune response, adaptive immune cells continue to infiltrate the CNS and remain there for several months. The whole-body inflammatory response of stroke patients not only reshapes the pathological outcome of stroke but also affects long-term outcomes by remodelling the immune system.^54^^,^^56^ (Fig. 3).

Shi et al. reported that a stroke-induced inflammatory response can occur and persist throughout the entire brain rather than being localized to the injured region.^7^ Emerging evidence suggests that immune cells not only interact with NVU cells in the brain but also become activated by brain-derived DAMPs or antigens and are closely regulated by peripheral neurotransmitters (Fig. 2). The miracle of the immune response during stroke lies in the fact that cerebrovascular infarction not only causes a strong local inflammatory response but also triggers systemic immunosuppression, especially an adaptive immune response in the periphery.^40^ Considering that neuroimmune interactions occur in both cerebral infarction and peripheral immune organs in stroke patients, we believe that intervention in peripheral immunity may also improve stroke outcomes; this is also supported by basic research and phase II clinical data.

Stroke is not merely a vascular condition or a consequence of neuronal death; it is also an immune-mediated disease. A variety of immune-related therapies have shown great promise after vascular recanalization,^11^, ^12^, ^13^ which highlights the pivotal involvement of maladaptive immunity in stroke pathogenesis. Therefore, the VIN reveals that stroke outcomes are governed by real-time crosstalk among the neurovascular unit, systemic immunity, and CNS-resident glial networks. The proposal of the VIN paradigm aims to inspire further research into the detailed mechanisms underlying stroke immunology.

## Immune-related brain cytoprotective therapy

While intravenous thrombolysis and endovascular thrombectomy have transformed AIS management, these reperfusion strategies remain applicable in merely 5–10% of patients owing to strict time windows during which these treatments are applicable. Notably, even among eligible patients, no more than 50% achieve functional independence postrecanalization, which is attributed to the “futile recanalization” phenomenon and the absence of adjunctive neuroprotective agents.^4^ This highlights the imperative for a dual strategy that combines revascularization with cytoprotection; this paradigm is poised to enhance penumbral preservation while mitigating reperfusion-associated sequelae (e.g., hemorrhagic conversion, vasogenic oedema, and systemic inflammatory complications).^2^^,^^11^^,^^13^

In combination with reperfusion therapies after stroke, many previously developed immune-related drugs, including the toll-like receptor 4 (TLR4) antagonist ApTOLL,^11^ methylprednisolone,^13^ edaravone dexborneol,^12^ fingolimod (FTY-720),^14^ and bone marrow mononuclear cell transplantation,^60^ have yielded promising results. Therefore, the immune system, which was once thought to primarily perform a medical function, has increasingly received researchers’ attention in the context of pharmacological drug development to treat stroke. To date, anti-inflammatory therapies, therapies that prevent immune cell infiltration or recirculation and cell-based therapies have been tested in stroke patients (Table 1, Table 2).

**Table 1:** Immune related cytoprotective agents have been evaluated in clinical trial.

Cytoprotective drug	Mechanism of action	Citation
Methylprednisolone	Immunosuppressive properties	Yang Q.W.^13^
ApTOLL	a DNA aptamer, is an antagonist at toll-like receptor 4 (TLR4)	Hernández-Jiménez. M.^11^
Bone marrow mononuclear cells	Immunomodulation, cell replacement, cell differentiation, neural circuit reconstruction, and protective factor release	Moniche.f.^60^
Colchicine	Inhibiting neutrophils migration and anti-inflammation	Li J.J.^61^
UK-279,276	A recombinant glycoprotein with selective binding to the CD11b/CD18	Krams M^62^
Natalizumab	Binding to VCAM-1 and CS-1 of fibronectin	Elkins J.^63^
Anakinra	IL-1 receptor antagonist	Cliteur MP.^64^
FTY-720	A sphingosine analogue that acts on sphingosine-1-phosphate receptors	Zhu Z.L.^14^
Edaravone Dexborneol	Anti-inflammatory and anti-oxidant drug	Fu Y.^12^

**Table 2:** Ongoing clinical trials of immune-related cytoprotective agents in stroke.

Cytoprotective drug	Public title	Date of Registration	Country
Fingolimod	Effect of Fingolimod in acute ischaemic stroke	2022-05-29	Iran
	Revascularization pretreated with fingolimod in acute stroke	2021-01-17	China
	Combinating fingolimod with alteplase bridging with thrombectomy in acute ischaemic stroke	2020-12-16	China
	Fingolimod in endovascular treatment of ischaemic stroke	2020-11-11	China
Colchicine	The fifth INTEnsive pReventing Secondary injury in acute cerebral haemorrhage trial within ACT-GLOBAL INTERACT5	2024-12-17	Australia China
	RIISC THETIS evaluation of low dose colchicine and ticagrelor in prevention of ischaemic stroke in patients with stroke due to atherosclerosis.	2024-08-27	France
	ACT-GLOBAL adaptive platform trial for stroke	2024-02-04	Australia Canada
	Efficacy of colchicine in preventing recurrent stroke in the patients with acute atherothrombotic ischaemic stroke during hospitalization COLCHIDA	2023-10-22	Russian Federation
	Evaluation of low dose colchicine and ticagrelor in prevention of ischaemic stroke in patients with stroke due to atherosclerosis RIISC-THETIS	2022-07-25	France
	Evaluation of low dose colchicine and ticagrelor in prevention of ischaemic stroke in patients with stroke due to atherosclerosis. “Reducing inflammation in ischaemic stroke with colchicine (riisc), and ticagrelor in high-risk patients-extended treatment in ischaemic stroke (thetis”)	2022-07-18	France
	Colchicine in high-risk patients with acute minor-to-moderate ischaemic stroke or transient ischaemic attack (CHANCE-3)	2022-06-20	China
	The effect of colchicine on cardiovascular outcomes in stroke study (The CASPER Study)	2021-10-20	Australia
	Effect of Colchicine on secondary prevention of ischaemic stroke	2020-06-21	Iran
Interleukin-6 receptor	Interleukin-6 receptor inhibition for symptomatic intracranial atherosclerosis IRIS-sICAS	2024-03-06	China
Methylprednisolone	Multicenter, randomized, controlled clinical trial on early combination therapy with methylprednisolone sodium succinate following acute large vessel occlusion recanalization.	2025-05-15	China
	Recanalization and acute perfusion improvement through intra-arterial delivery of methylprednisolone	2025-05-01	China
	Methylprednisolone with endovascular thrombectomy for large ischaemic stroke MATCH	2025-03-06	China
	Methylprednisolone as adjunct to endovascular thrombectomy for acute posterior circulation large vessel occlusion stroke: a multicenter, randomized, open-label clinical trial	2025-03-03	China
	Methylprednisolone adjunctive to endovascular treatment for stroke	2024-04-17	China
	Focal cerebral arteriopathy steroid trial FOCAS	2023-08-09	United States
	A prospective multicenter cohort study of early methylprednisolone treatment in acute stroke	2021-09-30	China
Glucocorticoids	Glucocorticoids as adjunct to endovascular thrombectomy for posterior circulation large vessel occlusion stroke: a multicenter prospective cohort study	2025-04-10	China
Dimethyl fumarate	Combination of the immune modulator dimethyl fumarate with alteplase in acute ischaemic stroke	2021-05-11	China
CRP-aphaeresis	Selective depletion of C-reactive protein by therapeutic aphaeresis (CRP-aphaeresis) in ischaemic stroke	2020-03-13	Germany
	CASTRO-B - study on CRP aphaeresis in STROke patients in berlin CASTRO-B	2019-03-19	Germany

### Anti-inflammatory therapies

The robust inflammatory response within the brain is a key mechanism that contributes to neuronal damage. Therefore, anti-inflammatory strategies have become the most extensively studied approach to poststroke immunotherapy. The interleukin-1 (IL-1) receptor antagonist anakinra has been tested in patients with cerebral haemorrhage and has shown potential to ameliorate neuroinflammation.^64^ Tocilizumab, which is a monoclonal antibody targeting the IL-6 receptor, has been approved for the treatment of multiple inflammatory diseases^65^ and is currently being tested in ongoing trials involving AIS patients (NCT06238024). Our previous data^66^ have shown that targeting TLR4 may lead to beneficial results after stroke. A phase 1/2 randomized clinical trial^11^ showed that ApTOLL was associated with meaningful clinical effects on AIS, including reducing mortality and disability at 90 days. In addition to specific targeted agents, multitarget agents are promising. Compared with edaravone alone, the multitarget drug edaravone dexborneol, which is composed of the antioxidant drug edaravone and the anti-inflammatory agent (+)-borneol, has been shown to be a superior cytoprotective agent.^12^ Sublingual edaravone dexborneol could also increase the proportion of patients with favourable functional outcomes at 90 days after AIS.^12^ There is no evidence to suggest that colchicine, despite its broad anti-inflammatory effects, reduces the risk of subsequent stroke within 90 days,^61^ but some multitargeted agents are still showing promise for AIS treatment. Glucocorticoids constitute another class of powerful immunosuppressants that can regulate the three sequential phases of the inflammatory response and promote the phagocytosis of apoptotic cells and debris by monocytes/macrophages.^67^ Although methylprednisolone did not reduce the number of deaths or improve functional outcomes of stroke patients before the advent of mechanical thrombectomy as a viable treatment option, our recent results showed that methylprednisolone can exert a protective effect on stroke when combined with reperfusion.^13^ Treatment with methylprednisolone can significantly decrease the rates of symptomatic intracranial haemorrhage within 48 h and mortality at 90 days.^13^ Therefore, more specific or multitarget anti-inflammatory agents should be tested or developed for the treatment of stroke.

### Therapies that block immune cell infiltration

The infiltration of immune cells into the brain predicts CNS damage caused by the immune response. Therefore, preventing immune cells from interacting with endothelial cells, thereby blocking their migration across the BBB into the CNS, is also considered a therapeutic strategy.^21^ Two adhesion blockers, namely, enlimomab and UK-279,276 (neutrophil inhibitory factor),^62^ have been tested in AIS patients following the advent of reperfusion therapies. Although these agents are well tolerated in AIS patients, the outcomes were disappointing. Natalizumab, which is a monoclonal antibody that targets α integrin within the adhesion molecule very late antigen-4 (VLA-4) to reduce the transmigration of leucocytes across the vascular endothelium, was also tested in combination with tPA.^63^ Unfortunately, natalizumab does not reduce infarct growth.^63^ The primary reason for this may be that vascular endothelial cells are severely damaged during the acute phase of stroke, making it difficult to achieve optimal therapeutic effects by targeting adhesion-related pathways in vascular endothelial cells.

### Therapies that prevent immune cell recirculation

After a stroke, a significant influx of peripheral immune cells, particularly those from the bone marrow, infiltrates the CNS,^38^ suggesting that blocking the mobilization of peripheral immune cells may offer an alternative solution. Fingolimod (FTY-720) blocks lymphocytes from exiting the lymph node. Therefore, it has been widely used in clinical practice for the treatment of multiple sclerosis.^68^ Encouragingly, FTY-720 has shown promising results in stroke treatment as well. A systematic review and meta-analysis of the efficacy of FTY-720 in animal models of stroke demonstrated its potential to reduce infarct volume and improve functional outcomes.^69^ Clinical pilot trial data have shown that oral fingolimod, when administered within 72 h of disease onset, can limit secondary tissue injury, decrease BBB permeability, attenuate neurological deficits, and promote recovery.^70^ When combined with alteplase treatment, oral fingolimod also attenuated reperfusion injury and improved clinical outcomes among AIS patients.^14^ Owing to its lack of specificity, FTY-720 can interact with multiple S1PRs, including S1P1 and S1P3, which are widely expressed in neurons and astrocytes.^68^ As a result, FTY-720 can also induce many side effects, such as microcystic macular oedema, lymphopenia, and transient dose-dependent bradycardia.^68^ Thus, the therapeutic efficacy of more selective S1P receptor modulators, such as ponesimod, etrasimod, or SEW2871, in AIS needs to be evaluated.^68^ Furthermore, the development of more specific monoclonal antibodies targeting S1P1 or new targets, such as spinster homologue 2 (SPNS2),^71^ which is an S1P transporter that enables effector T cells to egress from the lymph node, should be encouraged.

### Cell-based therapies

Under physiological conditions, the immune system maintains homoeostasis by balancing proinflammatory and anti-inflammatory responses. Therefore, a promising strategy is to normalize inflammatory injury by enhancing or promoting the anti-inflammatory response. After ischaemic stroke, infiltrating Treg cells have been reported to suppress neurotoxic astrogliosis by producing amphiregulin^41^ or promoting microglia-mediated white matter repair.^72^ In vitro data suggest that the transfer of allogeneic Treg cells^41^ or specifically increasing brain-resident Treg cell numbers with an interleukin-2 (IL-2):IL-2 antibody^72^ or astrocyte-targeted gene delivery of IL-2^73^ can protect against neuroinflammation and improve long-term stroke recovery. However, different subsets of IL-2-expanded human Treg cells have distinct functional and tissue-homing characteristics. Therefore, identifying the protective Treg cell subclass may be a promising therapeutic strategy.

As the origin of immune cells, the bone marrow inherently has the ability to control immune overactivation, such as myeloid-derived suppressor cells (MDSCs) in tumour immunity.^74^ Similarly, bone marrow mononuclear cell (BMMNC) transplantation has been developed as an alternative cell therapy to control inflammation in AIS patients.^60^ A phase 2 trial of intra-arterial delivery of BMMNCs in AIS patients did not show efficacy at the primary endpoint. However, secondary analyses suggested the potential for improved outcomes in moderate-to-severe stroke patients, including those undergoing thrombectomy.^60^ Although the mechanism is not well understood, the immunomodulatory effects of BMMNCs have been well documented.^75^ Moreover, BMMNC transplantation may also improve stroke recovery by modulating the peripheral immune system via interactions with splenocytes.^75^

## Conclusions and future directions

The important role of the immune system throughout the entire course of stroke has been recognized for a long time. However, the collaboration and mutual influence of the vascular, immune, and neural systems, especially their coordinated response after stroke, are not well understood. Thus, we propose the VIN paradigm, which is based in existing theories such as the NVU or VEN, to highlight the interaction between the immune and vascular systems, as well as the nervous system. Moreover, as recently recommended at STAIR,^4^ immunotherapy has demonstrated great potential for improving stroke outcomes both before and after perfusion when combined with revascularization, which is the cornerstone of stroke treatment.

Although we proposed the VIN paradigm, many scientific questions related to stroke immunity still urgently need to be solved. Systematic investigations of the changes in various immune cells across different tissues, particularly immune organs, at different time points poststroke are lacking. Immune inflammation is a double-edged sword, and its protective and damaging effects at different stages of stroke need to be accurately distinguished. The mechanisms underlying the prolonged infiltration of adaptive immune cells into the brain during the chronic phase of stroke warrant further investigation. It remains to be determined whether infiltrating adaptive immune cells promote nerve regeneration in the chronic stage of stroke.

In conclusion, immunotherapy shows great promise for stroke. However, more in-depth investigations are required to clarify the specific roles of various immune cells in different periods to achieve precise immunotherapy in the future.

## Outstanding questions

Although we have attempted to elucidate the landscape of stroke immunity in this article, owing to the insufficient depth and abundance of existing related research, the panorama of the role of the immune system in stroke still requires further exploration and summary. Furthermore, the lack of a comprehensive understanding of the immunological mechanisms of stroke hinders the effective combination of immunotherapy and recanalization therapy to improve patient prognosis, which consequently accounts for the failure of numerous previous clinical trials. Additionally, in the context of poststroke rehabilitation, the incorporation of immunotherapy, as opposed to relying on physical rehabilitation alone, may offer therapeutic prospects.

**Search strategy and selection criteria.**

References for this Review were identified by searches of PubMed between 2000 and January 2025, and references from relevant articles. The search terms “Stroke”, “ischaemic infarction”, “Acute ischaemic stroke”, “AIS”, “Hemorrhagic Stroke”, “brain injury”, “Central nervous system”, “Immune”, “Immune response”, and “Inflammation” were used. There were no language restrictions. The final reference list was generated on the basis of relevance to the topics covered in this Review.

## Contributors

Q.Y. conceived the project. Y.F., and Z.Z. researched data for the article. All authors contributed substantially to discussion of the content, wrote the article, and reviewed and/or edited the manuscript before submission. All authors read and approved the final version of the manuscript, and ensure it is the case.

## Declaration of interests

All authors declare no competing interests.
